# The Young Ones, pre-post results of a transdiagnostic ACT-training for young adults with Severe Mental Illness

**DOI:** 10.1007/s10597-025-01564-8

**Published:** 2026-03-07

**Authors:** K. L. Helmus, J. D. Vink, F. Schirmbeck, M. B. de Koning, I. Germeys, L. de Haan

**Affiliations:** 1 Department of Psychiatry, Amsterdam University Medical Centre and Dr. Bosman clinic, Meibergdreef, Amsterdam, the Netherlands; 2 Department Psychiatry, Amsterdam University Medical Centre, Amsterdam, Netherlands; 3 Dr. Bosman, Amsterdam, Netherlands; 4 Department of Public Mental Health, Central Institute of Mental Health, Medical Faculty Mannheim, Heidelberg University, Mannheim, Germany; 5 Arkin Institute for Mental Health, Amsterdam, Netherlands; 6 Faculty of Medicine of the Catholic University of Leuven, Head of Contextual Science Psychiatry, Louvain, Belgium

**Keywords:** Severe mental illness, Young adults, Transdiagnostic, Acceptance and commitment therapy, Group therapy

## Abstract

Young adults with severe mental illness (SMI) often face significant barriers to accessing mental health care, including (self)-stigmatization, social withdrawal, and a lack of appropriate treatment programs. These challenges underscore the need for recovery-oriented interventions tailored to the developmental needs of this population. The aim of this study was to evaluate Acceptance and Commitment Therapy (ACT), with its focus on psychological flexibility and values-based action, for young adults with SMI. This pre-post pilot evaluation assessed the indications for feasibility, and preliminary outcomes of *The Young Ones* (TYO), an eight-week, ACT-based, transdiagnostic group intervention designed for young adults with SMI. Outcomes included personal recovery, functional impairment, psychological flexibility, and self-stigmatization. To evaluate change over time, we conducted separate Linear Mixed Models (LMM) for each outcome measure using an intention-to-treat (ITT) approach. Of 101 eligible participants, sixty-one individuals enrolled in the research, and 33 completed both pre- and post-measures (46% attrition). Significant improvements were observed in personal recovery and functional impairment. No significant changes were found for psychological flexibility or self-stigmatization. TYO appears feasible and acceptable for young adults with SMI, with promising indications of benefit in personal recovery and daily functioning. While dropout remains a concern, these preliminary findings support the need for a randomized controlled trial to rigorously evaluate the effectiveness of ACT-based group interventions for this population.

## Introduction

### Young people with severe mental illness

Worldwide, mental health conditions are among the leading causes of disability and death in young adults, with depression, anxiety, and self-harm ranking among the main contributors to global disease burden in this age group (GBD 2019 Mental Disorders Collaborators, 2022; Erskine et al., 2017; WHO, 2023a). Suicide remains the third leading cause of death among 15–29-year-olds globally (WHO, 2023b), and young adults aged 18–25 report the highest prevalence of any mental illness (NIMH, 2023). Addressing mental health problems during this period can have a lifelong positive impact. Without treatment, symptoms can worsen to severe mental illness (SMI) (Gustavson et al., 2018). SMI is defined as a mental, behavioral, or emotional disorder that results in serious functional impairment and substantially interferes with or limits one or more major life activities (NIMH, 2023). Symptoms are often accompanied by severe social and societal limitations, persisting for several years (Folsom et al., 2005; Nordt et al., 2007). Young adults with SMI often face significant challenges in accessing care they require. Multiple studies and reports have highlighted the systemic barriers that contribute to this unmet need. A systematic review by de Beer et al. (2023) found that young adults with severe and enduring mental health problems frequently experience treatment dropout and ineffective care, often due to fragmented services and inadequate engagement strategies. Reasons for not receiving care include difficulties in securing appointments, cost-related issues, and a lack of available services in many areas. Stigma and self-stigmatization also remain pervasive barriers. The World Health Organization (WHO) notes that adolescents with mental health conditions are particularly vulnerable to social exclusion and discrimination, which can deter them from seeking help and enhance their self-stigmatization (Dubreucq et al., 2021; Gulliver et al., 2010), social withdrawal, and noncompliance (de Soet et al., 2023; Joe & Lee, 2016).

### Personalized recovery oriented care

The importance of diagnostic models and interventions tailored for young adults with severe mental illness (SMI) is increasingly recognized. Traditional categorical diagnostic systems often fail to capture the complexity and heterogeneity of mental health issues in this population. Recent research advocates transdiagnostic and developmental approaches that consider the multifaceted nature of psychopathology in young adults (Dalgleish & Black, 2020). These approaches emphasize the interplay of genetic, neurobiological, and environmental factors contributing to mental disorders, moving beyond one-dimensional disorder-focused models (Parkes et al., 2020). Such frameworks are particularly relevant for young adults undergoing significant social and emotional development, making the trajectory of symptom emergence and progression less predictable (McGorry et al., 2022). Adopting transdiagnostic multifactorial models facilitates early identification and personalized interventions, aligning with the evolving understanding of mental health in young adults. Transdiagnostic approaches are increasingly advocated in youth mental health care, particularly for young adults with severe mental illness (SMI), due to the high comorbidity, heterogeneity, and developmental complexity often observed in this population. These models offer a framework for addressing shared underlying processes across disorders rather than focusing on single diagnostic categories. In addition to better reflecting clinical realities, transdiagnostic interventions are often more cost-effective and easier to disseminate, as they allow clinicians to be trained in a unified approach that can be applied across diverse clinical presentations (Marchette & Weisz, 2017). This makes them particularly suitable for community mental health systems that serve youth with a wide range of intersecting needs.

Alongside new diagnostic understandings, more personal rather than solely clinical recovery-oriented therapies are being developed (Melillo, 2025). Clinical recovery includes remission of symptoms and functional improvement, whereas personal recovery refers to a personal process towards a productive and satisfying life in which people experience connectedness, hope, identity, meaning, and empowerment (CHIME) (Leamy et al., 2011). Personal recovery is recognized as an important part of the recovery process (Castelein, 2021; Leendertse et al., 2021; Van Eck et al., 2017) because symptoms often remain after treatment.

A paradigm shift is noticeable, and other interventions are emerging. Acceptance and Commitment Therapy (ACT) is one such intervention. ACT has a personal recovery-oriented approach. Instead of focusing on symptom reduction, patients are taught how to cultivate the ability to accept painful and troubling experiences, emotions, and thoughts and to commit to pursuing valued life goals and directions in the face of those experiences (Hayes et al., 2004).

### Acceptance and commitment therapy

ACT is a ‘third generation CBT’ approach, in which the focus lies on creating psychological flexibility. This rests on the fundamental premise that pain, grief, disappointment, (mental) illness, and stress are inevitable features of human life. The therapeutic aim is to help people adapt to these challenges by developing greater psychological flexibility instead of engaging in counterproductive attempts to completely eliminate or suppress undesirable experiences (S. C. Hayes et al.,^2006^). Using the ACT model, key mechanisms can support adaptive change by refining and emphasizing effective strategies while eliminating counterproductive strategies (S. C. Hayes et al., 2012). It contributes to psychological flexibility through processes such as acceptance (allowing experiences to occur), cognitive defusion (disengaging from stressful thoughts), contact with the present moment (non-judgmental awareness of what is being experienced in the moment), values (clarity about what is important to an individual), committed action (acting in accordance with personal values), and self-as-context (flexible perspective-taking skills) (A-Tjak et al., 2015). Recent systematic reviews and meta-analyses have examined the application of ACT in populations with severe mental illness, particularly psychotic disorders. For instance, Yıldız (2019) reviewed 11 randomized controlled trials and reported beneficial effects of ACT on depression, anxiety, and psychotic symptoms. However, a more cautious interpretation comes from Brown et al. (2021), whose meta-analysis concluded that while ACT appears promising, the current evidence base has methodological limitations. Nevertheless, broader reviews, such as Gloster et al. (2020), support ACT’s transdiagnostic effectiveness, with a range of small to large effects reported across various psychiatric conditions. These findings suggest that ACT holds significant potential as a recovery-oriented intervention for individuals with severe mental illness, particularly in alignment with personal recovery goals. Self-stigmatization, loneliness, and challenges in identity formation are significant concerns among young adults with severe mental illness (SMI), often hindering their recovery process. Interventions that normalize mental health experiences and emphasize shared human struggles can effectively reduce self-stigmatization. Acceptance and Commitment Therapy (ACT) has been shown to decrease stigmatizing attitudes by transforming 'us versus them' thinking into continuum beliefs, thereby reducing self-stigmatization among patients (Pinfold, Thornicroft, Huxley, & Farmer, 2005).

### ACT group intervention

Peer support within group settings has been identified as a valuable component in mental health care, providing individuals with lived experiences the opportunity to support each other, which can alleviate feelings of isolation and promote a sense of belonging (Repper & Carter, 2011). These peer connections are particularly beneficial for young adults, as they contribute to socialization and identity development during a critical period of personal growth (Turner & Shepherd, 2015). The core ACT processes lend themselves well to delivery in group formats, where shared experiences, interpersonal learning, and social modeling can enhance therapeutic engagement and reinforce behavioral change. Group-based ACT interventions not only provide a cost-effective means of treatment delivery but also create opportunities for peer learning and mutual support.

While research specifically examining the effects of group-based ACT interventions on young adults with SMI is limited, research suggests that such approaches, which combine the principles of ACT with the benefits of peer support, hold promise addressing the unique challenges faced by this population (Hayes, Strosahl, & Wilson, 2004). There also remains a notable gap in research concerning the application in transdiagnostic group-based interventions specifically tailored for young adults with *severe mental illness* (SMI). The feasibility and acceptability of ACT group intervention for people with psychosis have shown improvements in clinical outcomes (Johns et al., 2016), and an inpatient study with a group ACT intervention showed improvement in personal recovery measures (Jeong, Jeon, You, & Lee, 2022). Preliminary studies have demonstrated the feasibility and acceptability of group-based ACT interventions in adolescent populations, with evidence of improvements in psychological flexibility and reduction in psychological symptoms (Livheim et al., 2015).

### The Young Ones

Building on the emerging evidence for ACT, we developed and implemented "The Young Ones," (TYO) an eight-week, group-based ACT intervention for young adults, grounded in the DNA-V model, adjusted for the Dutch mental health context. We developed this intervention since group-based interventions are scarce for young adults aged 18–35 with SMI. Moreover this group is substantial, about 2% of the youth between age 18 and 35 meet the criteria for SMI (Delespaul, 2013; Kroon H., 2016; NIMH, 2023). The DNA-V model is a framework that integrates ACT principles with developmental psychology and positive psychology to enhance psychological flexibility among young adults (Hayes & Ciarrochi, 2015). This model focuses on cultivating skills such as self-awareness, value-driven action, and adaptive social behaviors, making it particularly suitable for adolescents and young adults navigating complex mental health challenges. It emphasizes experiential learning and promotes flexible identity development through three functional repertoires: the Discoverer, the Noticer, and the Advisor, guided by personal values. This model is particularly suited for youth with SMI due to its focus on psychological growth, autonomy, and social connection (Ciarrochi et al., 2016).

We discuss TYO intervention in depth in the method section of this paper. This study is the quantitative part of a mixed-methods pilot study aimed to assess the feasibility and preliminary evaluation of the effectiveness of *The Young Ones* for this population, contributing to the limited but growing body of research on ACT interventions for young adults with SMI. This paper presents the feasibility and preliminary evaluation of the pre-post results. We expected to find insights into the feasibility and potential impact of the intervention on increased scores on personal recovery, decrease in functional impairment, self-stigmatization, and enhanced psychological flexibility.

## Method

### Treatment setting

This study was conducted in a routine clinical care context within specialized mental health services in Amsterdam, targeting young adults diagnosed with severe mental illness (SMI). The Acceptance and Commitment Therapy (ACT)-based group intervention, *"The Young Ones"* (TYO), was initially developed and implemented several years prior by two of the authors, who are both clinicians and researchers, alongside other colleagues and patients, in response to the need for age-appropriate, recovery-oriented interventions for young adults with severe mental illness. Although TYO had been offered across several outpatient and community-based mental health teams, it had not yet been formally evaluated. The participating teams included two specialized outpatient teams for young adults with psychosis of the mental health setting Mentrum, an inpatient psychosis treatment clinic of a hospital (Amsterdam Medical Centre), and two assertive community treatment teams also part of Mentrum.

### Participants

To participate in TYO, patients had to meet the following criteria:

- age between 17 and 37 years
- a clinical diagnosis of a mental disorder classified according to the Diagnostic and Statistical Manual of Mental Disorders, Fifth Edition (DSM-5; American Psychiatric Association, 2013)
- meeting the Dutch consensus criteria for SMI; the presence of a psychiatric disorder combined with enduring functional impairments and significant limitations in societal participation (Delespaul, 2013)
- willingness and ability to join a group-based intervention
- sufficient proficiency in Dutch language

During the eight-week intervention period in which TYO was delivered, the participants continued receiving regular treatment. This typically consisted of pharmacological treatment (85% of participants were prescribed psychiatric medication) and regular contact with mental health professionals, with 95% of participants having attended at least one appointment in the four weeks preceding the intervention. TYO was offered as add-on intervention to their regular treatment.

### Study design and procedure

The study design is a pre-post evaluation with two timepoints: pre-treatment (T1) and immediately post-treatment (T2). Written informed consent was obtained prior to session one. Participants were included in the analysis if they attended a minimum of four sessions and completed both pre- and post-assessments. The study protocol was reviewed and approved in accordance with Dutch medical research regulations by the Medical Research Ethics Committee of the Amsterdam Medical Center (reference: NL70317.018.19) as part of a mixed-method study. Information about the study was provided to eligible participants during routine care and they were invited to participate voluntarily in the Young Ones and in the study.

### Intervention

*The Young Ones* (TYO) intervention was originally developed specifically for young people with SMI by members and patients of the clinical and research team, drawing upon established ACT frameworks and adapting them to suit the developmental needs and clinical complexity of young adults with severe mental illness (SMI). The core structure and conceptual foundation of the intervention were based on the DNA-V model (Ciarrochi et al., 2016).

While DNA-V has previously been applied in adolescent settings (Hayes & Ciarrochi, 2015), *The Young Ones* represents a novel application of the model tailored for young adults with SMI receiving care in outpatient and community mental health settings. The program was informed by materials and exercises from ACT for psychosis (Bach & Hayes, 2019) and DNA-V resources for youth. The intervention was already implemented and consisted of eight weekly, closed-group sessions (10–15 participants), each lasting three hours, co-facilitated by one or two trained ACT therapists. Sessions focused on experiential exercises that encouraged psychological flexibility and real-life skill application. Each session followed a structured format including psychoeducation, values exploration, mindfulness, defusion techniques, and behavioral activation. To promote engagement and reduce stigma, therapists used self-disclosure, group discussion, and collaborative exercises.

A key experiential component of TYO was shared meal preparation, conducted in pairs before each session. This activity functioned as an applied DNA-V practice: participants used the Advisor to plan, the Discoverer to engage and try new skills, and the Noticer to experience the present moment. The meals provided a naturalistic setting for practicing ACT processes, fostering peer connection, social interaction, and the embodiment of values such as cooperation, care, and creativity.

Participants received a client handbook with worksheets to reinforce learning between sessions. Group reminders were used to share homework tasks, and support continuity between sessions. Table 1 provides an overview of the intervention content across the eight weeks.

**Table 1:** The Young Ones program

Session	Session content
1. Acceptance	● psycho-education: 3 happiness myths (video) ● exercise: introducing yourself (exercise) ● psycho-education: happiness trap (video) and emotions ● exercise: feeling your emotions ● psycho-education: DNA-V method homework: do something outside your comfort zone
2. Values	● psychoeducation: values, value-based vs. goal-based living (video), values as compass for behavior ● exercise: what would you do if you would win a lottery? ● exercise: choose your value homework: take a picture of your value
3. Adviser	● psycho-education: Adviser, sushi train (video) ● exercise: defusion by singing the Adviser self-talk ● exercise: give your Adviser a name ● exercise: defusion exercise homework: observe your Adviser
4. Noticer	● psycho-education: Noticer ● psycho-education: mindfulness ● exercise: singing ‘Let it be’ ● exercise: eating chocolate ● exercise: BOLD: breath, observe, listen to your values, decide and take action homework: perform an everyday task mindfully
5. Discoverer	● psycho-education: Discoverer ● exercise: ‘I would like to…’ questionnaire ● exercise: setting goals ● exercise: explore your different Discoverer modes homework: undertake something with your Discoverer
6. Reflection	● psycho-education: DNA-V method ● psycho-education: 3 happiness myths (video) ● exercise: feeling your emotions ● psycho-education: mindfulness (video) ● exercise: BOLD homework: take a picture of your value
7. Self-compassion and open session	● exercise: feeling your emotions ● psycho-education: self-compassion (video) ● free choice of exercise homework: be kind to yourself
8. Closing session	● exercise: group poster creative project to visualize learnings of the past eight weeks going to a restaurant for dinner together

### Outcome Measurements

#### Demographics and feasibility

Demographic characteristics including age, gender, and primary DSM-5 diagnoses were retrieved from the electronic patient database (EPD), for which participants provided informed consent. Diagnoses were coded by clinicians as part of routine clinical documentation. Feasibility was anonymously assessed using the following quantitative indicators: 1. percentage of participants who attended at least one session, 2. percentage of participants who completed at least four sessions and 3. percentage of participants who completed all sessions. While no standardized feasibility questionnaire was administered, attendance, retention, and participant uptake were used as proxy indicators.

#### Instruments

The following instruments for the pre-post measurements were selected based on their alignment with ACT principles and recovery-oriented focus. The selection was theory-driven and designed to assess changes in personal recovery, functioning, self-stigma, and psychological flexibility, all central constructs in the ACT model and relevant to individuals with SMI.

Mental Health Recovery Measure (MHRM-NL): The MHRM-NL is a 30-item self-report measure developed to assess personal recovery and has been validated for use in intervention studies to track change over time (Van Nieuwenhuizen et al., 2013). It evaluates eight dimensions: overcoming stuckness, self-empowerment, learning and self-redefinition, basic functioning, overall well-being, new potentials, advocacy/enrichment, and spirituality. Items are scored on a 5-point Likert scale ranging from 1 (“strongly disagree”) to 5 (“strongly agree”), with higher scores indicating greater recovery. The total score ranges from 30 to 150. The MHRM-NL has demonstrated strong psychometric properties, including Cronbach’s α between .86 and .94. It has previously been used to evaluate recovery-oriented interventions in clinical settings.

Internalized Stigma of Mental Illness Scale (ISMI-9): The ISMI-9 is a brief 9-item version of the original ISMI-29, designed to measure internalized stigma among people with psychiatric disorders (Hammer & Toland, 2017). Each item is rated on a 4-point Likert scale from 1 (“strongly disagree”) to 4 (“strongly agree”), with higher scores indicating more severe self-stigma. Sample items include: “I can have a good, fulfilling life, despite my mental illness” and “I feel out of place in the world because I have a mental illness.” The total score is calculated as the mean item score and interpreted using a four-tier system (Lysaker et al., 2007): 1.00–2.00 (minimal to no stigma), 2.01–2.50 (mild), 2.51–3.00 (moderate), and 3.01–4.00 (severe). Cronbach’s α in our study was .86.

Flexibility Index Test (FIT-60): The FIT-60 is a 60-item self-report questionnaire designed to assess psychological flexibility, the core process targeted by ACT (Batink et al., 2012; 2015). It consists of six subscales aligned with ACT processes: acceptance, cognitive defusion, self-as-context, contact with the present moment, values, and committed action. Each item is rated on a 7-point Likert scale from 0 (“completely disagree”) to 6 (“completely agree”). Example items include: “I observe my feelings without losing myself in them” and “I don’t always have to do things right.” Higher scores indicate greater psychological flexibility. The FIT-60 demonstrates excellent psychometric properties (Cronbach’s α = .90).

Sheehan Disability Scale (SDS): The SDS is a brief, 3-item measure of functional impairment in work/school, social life, and family life (Sheehan et al., 1996). Each domain is rated on an 11-point scale (0 = “not at all impaired” to 10 = “extremely impaired”). Mean item scores were calculated, and severity was categorized as mild (1–3), moderate (4–6), marked (7–9), or severe (10). An example item is: “In the past week, how much have your symptoms disrupted your ability to do well at work or school?” The SDS has an excellent test–retest reliability (ICC = .98) and internal consistency (Cronbach’s α = .89).

These outcome measures align closely with the theoretical foundations of Acceptance and Commitment Therapy (ACT) and the goals of recovery-oriented care. The MHRM-NL captures personal recovery dimensions such as empowerment, meaning-making, and well-being, which resonate with ACT’s focus on values and committed action. The ISMI-9 addresses internalized stigma, a key barrier to recovery. By measuring changes in self-perception ACT is thought to target self-perception through two mechanisms: cognitive defusion and self-as-context. The FIT-60 assesses psychological flexibility, the core mechanism of change in ACT, across six key processes. Lastly, the SDS evaluates real-world functional impairment, providing insight into how therapeutic gains manifest in daily life. Together, these instruments offer a comprehensive evaluation of both processes and outcomes central to ACT and recovery-focused interventions for young adults with SMI.

### Data Analysis

Feasibility outcomes were assessed using descriptive statistics. For the pre-post measurements the data analyses were performed using IBM SPSS Statistics, Version 24. First, the dataset was screened for missing values, outliers, and normality using Q-Q plots (Kang, 2013). Descriptive statistics (means, standard deviations, ranges, and frequencies) were calculated to summarize demographic variables and attendance. To evaluate potential baseline differences between participants who completed the intervention (defined as attending ≥ 4 sessions and completing both pre- and post-treatment measures) and those with incomplete participation or missing post-assessments, we conducted a one-way Analysis of Variance (ANOVA). The dependent variables were baseline scores on the four outcome measures: Mental Health Recovery Measure (MHRM-NL), Internalized Stigma of Mental Illness Scale (ISMI-9), Flexibility Index Test (FIT-60) and the Sheehan Disability Scale (SDS). Additionally, chi-square tests were used to examine group differences in gender and primary diagnosis. To evaluate change over time, we conducted separate Linear Mixed Models (LMM) for each outcome measure using an intention-to-treat (ITT) approach. LMM is suitable for analyzing pre-post data with incomplete observations, as it accommodates missing data under the assumption of Missing at Random (MAR) and accounts for repeated measures within individuals. Each model included: Time (pre- vs. post-treatment) as a categorical fixed effect (reference category: pre-treatment) random intercepts for both individual participants and treatment group (Groups 1–8) to account for clustering and within-subject variation. Unstructured covariance matrix to allow for flexible estimation of variances at each time point Restricted Maximum Likelihood Estimation (REML) for model fitting. This modeling approach allowed us to test whether outcome measures showed statistically significant changes from pre- to post-treatment, while adjusting for the nested design and incomplete data. A significance level of α = .05 was used for all statistical tests, and 95% confidence intervals were reported for all estimated effects.

## Results

### Feasibility

**Fig. 1:**
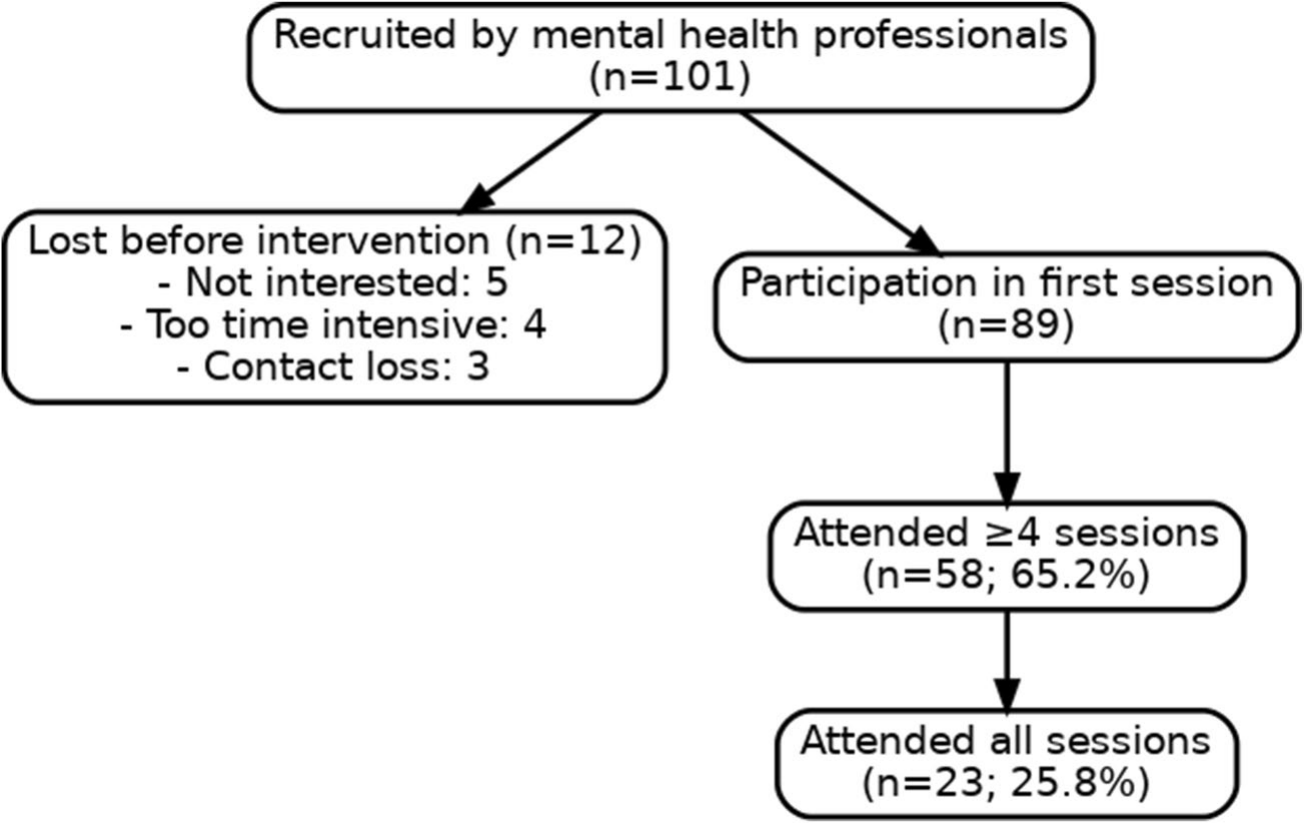
Flow-chart feasibility

A total of 101 eligible individuals were approached by mental health professionals affiliated with participating treatment teams. Of these, 88% (n = 89) attended at least one session of *The Young Ones* (TYO). Reasons for non-participation included anxiety about group treatment, scheduling conflicts, or lack of interest. A total of 58 attended at least four sessions and n = 23 attended all sessions of TYO (Fig. 1).

### Participant recruitment for the research

**Fig. 2:**
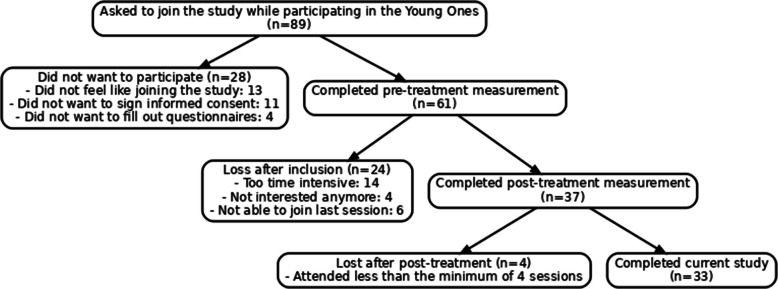
Flow chart study

Of the 89 participants who started TYO, 61 provided written informed consent and completed the pre-treatment assessment, thereby enrolling in the study. Post-treatment data were available for 37 participants. However, four participants attended fewer than four sessions, the minimum threshold to be considered as having received sufficient exposure to the intervention. Therefore, the final analytic sample consisted of 33 participants who completed both pre- and post-measurements and attended ≥ 4 sessions. The 24 participants that were lost after inclusion were classified as having incomplete participation or missing data. Reasons for non-completion included scheduling conflicts, lack of motivation, or not being able to join the last session (see Flow chart Fig. 2).

#### Group Format

Eight closed-format TYO groups were conducted during the study period. Each group consisted of 10–15 participants and was co-facilitated by one or two trained ACT therapists. Groups were not open; participants started and finished the intervention together. Study participants were mixed with non-participating clients from the same services who received TYO but opted out of the research.

#### Demographics attendance and primary diagnosis

Table 2 presents demographic information attendance and primary diagnosis data for the 61 study participants. Participants ranged from 19 to 36 years old (M = 27.4, SD = 3.96). The majority were diagnosed with a psychotic disorder, and almost all had comorbid DSM-5 diagnoses. The average number of sessions attended was 5.31 (SD = 2.56). Due to sample size limitations, we did not statistically compare outcomes across different session doses, but this is noted as a limitation and direction for future research. Nearly half (48%) attended at least 80% of sessions, and 30% completed all eight sessions.

**Table 2:** Demographic characteristics session attendance and primary diagnosis (n = 61) (*SD* = *standard deviation of session attendance*)

**Variable**		
Age (range 19-36)	M 27.4	SD 3.96
Sessions attended (range 1-8)	M 5.31	SD 2.56
Sessions attended	N	%
1	6	9.8
2	8	13.1
3	5	8.2
4	3	4.9
5	5	8.2
6	6	9.8
7	10	16.4
8	18	29.5
**Gender**		
**Male**	39	63.9
**Female**	22	36.1
**Primary DSM-5 diagnosis**		
Psychotic disorders	38	62.3
Bipolar disorders	6	9.8
Substance-related disorders	7	11.5
Neurodevelopmental disorders	3	4.9
Depressive disorders	2	3.3
Anxiety disorders	2	3.3
Avoidant personality disorder	1	1.6
Eating disorder	1	1.6
Missing diagnosis	1	1.6

### Descriptive Analyses

#### Baseline Differences by Completion Status

Table 3 shows comparisons between participants who completed the intervention (n = 33) and those with incomplete participation or missing post-data (n = 28). One-way ANOVA analyses found no significant differences in age or baseline scores on the MHRM, ISMI-9, FIT-60, or SDS between the two groups. Chi-square analyses showed no significant differences in gender ((χ^2^) = 0.10, p = 0.75) or primary diagnosis (χ^2^) = 1.88, p = 0.87.

**Table 3:** Baseline comparisons between completion groups

Variable	Participants with incomplete attendance/data (M, SD)	Participants with complete attendance and data (M, SD)	F(1, 59)	*p*
Age	27.18 (3.72)	27.64 (4.21)	0.20	.657
Recovery	102.10 (9.89)	102.48 (15.06)	0.00	.991
Self-Stigma	2.54 (0.67)	1.98 (0.46)	0.33	.566
Psychological Flexibility	197.24 (44.02)	201.89 (46.08)	0.16	.687
Functional Impairment	5.41 (2.56)	4.57 (2.41)	1.74	.192
Gender				
Male	19 (67.9%)	20 (60.6%)	0.35	.557
Female	9 (32.1%)	13 (39.4%)		
Primary diagnoses				
Psychotic disorders	18 (64.3%)	20 (60.6%)		
Bipolar disorders	3 (10.7%)	3 (9.1%)		
Substance-Related disorders	1 (3.6%)	6 (18.2%)		
Neurodevelopmental disorders	2 (7.1%)	1 (3.0%)		
Depressive disorders	0 (0.0%)	2 (6.1%)		
Anxiety disorder	1 (3.6%)	1 (3.0%)		
Avoidant personality disorder	1 (3.6%)	0 (0.0%)		
Eating disorder	1 (3.6%)	0 (0.0%)		
Missing diagnosis	2 (7.1%)	1 (3.0%)		

#### Preliminary Outcomes

Results from the Linear Mixed Model (LMM) analyses are presented in Table 4. LMM included all participants with at least one measurement and adjusted for clustering by treatment group and participant.

**Table 4:** Outcome Measures: Observed Mean and Standard Deviation and Estimated Change Based on the Estimates Derived from the Linear Mixed Model in an ITT Sample

**Outcome**	**Observed Mean (SD)**	Observed Mean	**Estimated Change (SE)**	***p***	**95% CI**	
	Pre-treatment	Post-treatment			LL	UL
MHRM	103.07 (16.88)	107.83 (14.55)	5.15 (1.80)	.007	1.62	8.68
ISMI	2.02 (0.56)	2.00 (0.48)	-0.02 (0.06)	.728	-0.11	0.08
FIT	195.49 (44.83)	201.89 (48.62)	7.03 (4.49)	.126	-1.75	15.81
SDS	4.82 (2.41)	3.90 (2.50)	-0.87 (0.40)	.033	-1.65	-0.09

Estimates represent model-based change from baseline to post-intervention derived from linear mixed-effects models with random intercepts, using restricted maximum likelihood estimation. CI = Confidence Interval, LL = lower limit, UL = upper limit, MHRM = Mental Health Recovery Measure (N = 60), ISMI = Internalized Stigma of Mental Illness (N = 61), FIT = Flexibility Index Test (N = 61), SDS = Sheehan Disability Scale (N = 59)

## Discussion

This study presents quantitative findings, as the study was part of a mixed-methods pilot evaluation of *The Young Ones* (TYO), a transdiagnostic, ACT-based group intervention tailored for young adults with severe mental illness (SMI). The primary aim was to assess feasibility, and preliminary effects on recovery-related outcomes within the context of specialized young adults mental health services.

### Feasibility

The findings indicate good initial engagement, with 88% of eligible individuals attending the first session, 65.2% attending at least four sessions, and 25.8% attending all sessions. The implementation of TYO across diverse clinical teams proved feasible and was well integrated into routine care pathways. This is consistent with literature suggesting that young adults with SMI are willing to engage with interventions that are accessible, peer-oriented, and experiential (WHO, 2008; IGJ, 2021). However, a substantial proportion of individuals (34.8%) attended fewer than four sessions. This level of attrition is comparable to dropout rates commonly reported in psychosocial interventions for youth with SMI, which often range between 25 and 75% (Bouchard et al., 2022; de Soet et al., 2023). This highlights a common challenge when working with young people facing motivational, cognitive, or practical barriers to sustained participation. To address this, future implementations could benefit from stronger pre-treatment motivational components, more flexible delivery formats, or incentive structures. Importantly, this attrition was not associated with baseline demographic or clinical characteristics.

### Preliminary effects

Statistically significant improvements were observed in personal recovery (MHRM) and functional impairment (SDS), suggesting that TYO may support domains relevant to self-agency, goal pursuit, and daily functioning, core targets of ACT and recovery-oriented care. These findings align with previous group-based ACT studies showing positive outcomes in similar domains (Jeong et al., 2022; Johns et al., 2016). The structure of TYO, particularly its values-oriented exercises, peer interactions, and experiential learning activities may have contributed to these improvements. However, no significant changes were observed in self-stigmatization (ISMI) or psychological flexibility (FIT-60). It is possible that eight sessions were insufficient to impact deeper cognitive and experiential processes like stigma internalization or psychological flexibility, which may require longer-term engagement or booster sessions. Furthermore, the FIT-60, though well-validated, may not be sensitive enough to detect subtle shifts over a short duration, especially in a heterogeneous population with complex needs.

### Intervention design considerations

TYO’s integration of shared meal preparation is one unique feature of the program. Rooted in the DNA-V model, this activity was designed to facilitate real-time practice of ACT principles in a low-stakes, social environment. It allowed participants to explore values (e.g., connection, creativity), defuse unhelpful self-talk (Advisor), experiment behaviorally (Discoverer), and mindfully engage in the moment (Noticer). Such structured experiential tasks can enhance generalization of learning and foster group cohesion, which are important mediators of therapeutic change in young adults (Leader, 1991; Hayes & Ciarrochi, 2015).

### Limitations

Several limitations must be acknowledged. First, this was a non-randomized, uncontrolled pre-post study, which limits causal interpretations and introduces the possibility of confounding variables influencing outcomes. Second, although qualitative interviews explored participant experiences (qualitative findings are reported elsewhere), the study did not include standardized quantitative measures of feasibility or acceptability, which limits comparability to other pilot evaluations. Third, the age range (19–36) extended beyond typical young adult classifications, potentially affecting generalizability. The study also relied on self-report measures, which may be subject to biases. Due to sample size limitations, we did not statistically compare outcomes across different session doses. While the meal preparation was thought to be a central experiential component of TYO, its specific contribution to outcomes was not measured and can be considered in future research. Finally, as an implementation within real-world care, TYO groups included participants from different settings without a standardized recruitment procedure. This may have influenced the composition of treatment groups. However, as said, this reflects regular care and may be viewed as a factor that increases generalizability of current findings.

### Future directions

This pilot provides a foundation for a more robust evaluation. Future studies could incorporate: A randomized controlled trial (RCT) design powered for small to medium effects; standardized measures of treatment acceptability and engagement; assessing outcomes related to the prevention of re-hospitalization, crisis situations, and social decline; a longer follow-up period to assess maintenance of effects, consideration of adaptations (e.g., digital components, flexible formats) to reduce dropout and process evaluations to explore mechanisms of change and treatment fidelity.

### Conclusion

This pilot study suggests that *The Young Ones*, an ACT-based group intervention grounded in the DNA-V model, seems feasible to implement in specialized mental health services for young adults with SMI. Preliminary findings indicate potential benefits for personal recovery and functional improvement. While no changes were found in self-stigma or psychological flexibility, the overall results support the value of further testing through a randomized controlled trial. This next step will be crucial in establishing the clinical effectiveness and implementation potential of TYO within young adults mental health care systems.
